# Myocardial deformation assessed by Longitudinal Strain: chamber-specific normative data for CMR-feature tracking from the German competence network for congenital heart defects

**DOI:** 10.1186/1532-429X-17-S1-P202

**Published:** 2015-02-03

**Authors:** Quanliang Shang, Shelby Kutty, David Danford, Michael Steinmetz, Andreas Schuster, Titus Kuehne, Philipp B Beerbaum, Samir Sarikouch

**Affiliations:** University of Nebraska Medical Center, Chiildren’s Hospital and Medical Center, Omaha, NE USA; University Medical Center, Göttingen, Göttingen, Germany; German Heart Institute, Berlin, Germany; Hanover Medical University, Hannover, Hannover, Germany

## Background

Two-dimensional longitudinal strain (LS) is a quantitative and automated technique for the measurement of global long-axis function in any cardiac chamber. Observational studies of left ventricular function in adults suggest that global LS correlate with EF, and is superior to EF as a predictor of outcome. Global LS can be derived by cardiac magnetic resonance (CMR), however its application in routine clinical practice is hampered by the lack of chamber-specific normative data. The purpose of this investigation is to provide reference data for atrial and ventricular global LS during childhood and adolescence by CMR feature tracking (FT).

## Methods

We prospectively enrolled healthy children and adolescents in participating centers of the German Competence Network for Congenital Heart Defects. CMR was performed on 1.5T scanners and consisted of a stack of standard two-dimensional steady-state free-precession (SSFP) acquisition covering the whole heart in the transverse plane. CMR-FT was performed on ventricular horizontal long axis SSFP images (4-chamber view, 6-segment model) for derivation of right and left atrial (RA, LA), and right and left ventricular (RV, LV) peak global LS (2DCPA-MR, TomTec, Germany). Atrial and ventricular ejection fractions (EF) and end diastolic volumes (EDV) were also measured (Medis, the Netherlands). Correlations were explored for LS with age, body surface area (BSA), gender, and EDV and EF of each chamber.

## Results

There were 115 subjects (male: female 56:59, mean age 12.4 ± 4.1 years; range, 4.4-20.3 years, BSA 1.4±0.4 m^2^). The mean ± SD of LS (%) for RA, RV, LA and LV were 26.6±10, 18±5, 26.5±10.6 and 17.5±5 respectively. There was significant positive correlation of LS in the LA, LV, RA and RV with corresponding EF (all p<0.05, Figure). Gender-wise differences (all p >0.05) were not significant for atrial and ventricular LS, and correlations with age and body surface area were generally weak. Inter and intra-observer comparisons in 20 randomly selected subjects showed good agreements for both atrial and ventricular LS.

**Figure 1:**
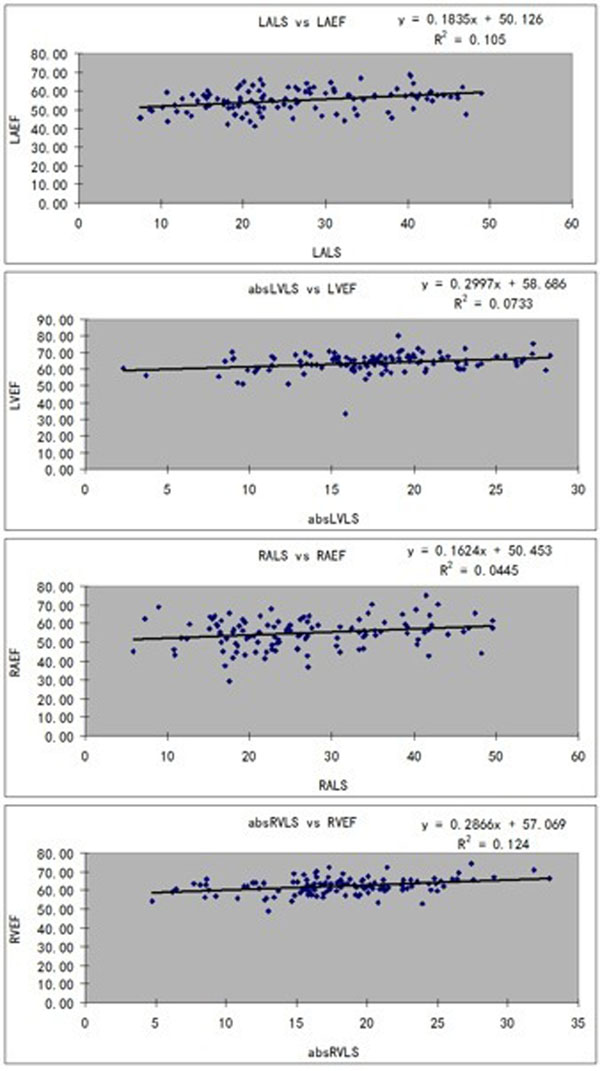
Figure 1

## Conclusions

This investigation provides chamber-specific nomograms for pediatric atrial and ventricular LS to serve as clinical reference, and to facilitate future CMR-based deformation research.

## Funding

German Competence Network for Congenital Heart Defects.

